# Silencing airway epithelial cell-derived hepcidin exacerbates sepsis-induced acute lung injury

**DOI:** 10.1186/s13054-014-0470-8

**Published:** 2014-08-06

**Authors:** Qi Xing Chen, Sheng Wen Song, Qing Hua Chen, Cong Li Zeng, Xia Zheng, Jun Lu Wang, Xiang Ming Fang

**Affiliations:** Department of Anesthesiology, the First Affiliated Hospital, College of Medicine, Zhejiang University, 79 Qing Chun Road, Hangzhou, 310003 China; Department of Anesthesiology, the First Affiliated Hospital of Wenzhou Medical University, 36 Gong Yuan Road, Wenzhou, 325000 China; Intensive Care Unit, the First Affiliated Hospital, College of Medicine, Zhejiang University, 79 Qing Chun Road, Hangzhou, 310003 China

## Abstract

**Introduction:**

The production of antimicrobial peptides by airway epithelial cells is an important component of the innate immune response to pulmonary infection and inflammation. Hepcidin is a β-defensin-like antimicrobial peptide and acts as a principal iron regulatory hormone. Hepcidin is mostly produced by hepatocytes, but is also expressed by other cells, such as airway epithelial cells. However, nothing is known about its function in lung infections and inflammatory diseases. We therefore sought to investigate the role of airway epithelial cell-derived hepcidin in sepsis-induced acute lung injury.

**Methods:**

Acute lung injury was induced by polymicrobial sepsis via cecal ligation and puncture (CLP) surgery. Adenovirus-mediated short hairpin RNA specific for the mouse hepcidin gene *hepc1* and control adenovirus were intratracheally injected into mice. The adenovirus-mediated knockdown of hepcidin in airway epithelial cells was evaluated *in vivo*. Lung injury and the seven-day survival rate were assessed. The levels of hepcidin-related iron export protein ferroportin were measured, and the iron content and function of alveolar macrophages were evaluated.

**Results:**

The hepcidin level in airway epithelial cells was upregulated during polymicrobial sepsis. The knockdown of airway epithelial cell-derived hepcidin aggravated the polymicrobial sepsis-induced lung injury and pulmonary bacterial infection and increased mortality (53.33% in Ad-shHepc1-treated mice versus 12.5% in Ad-shNeg-treated mice, *P* <0.05). The knockdown of hepcidin in airway epithelial cells also led to reduced ferroportin degradation and a low intracellular iron content in alveolar macrophages. Moreover, alveolar macrophages form the airway epithelial cell-derived hepcidin knockdown mice showed impaired phagocytic ability than those from the control mice.

**Conclusions:**

Airway epithelial cell-derived hepcidin plays an important role in CLP-induced acute lung injury. The severe lung injury in the airway epithelial cell-derived hepcidin knockdown mice is at least partially related to the altered intracellular iron level and function of alveolar macrophages.

## Introduction

Acute lung injury (ALI) and its severe form, acute respiratory distress syndrome (ARDS), are common clinical disorders and present substantial health problems worldwide [1-3]. The incidence of ALI has been reported to be 190,000 cases per year in the United States, and sepsis is one of the most commonly encountered conditions underlying the development of ALI [1,4]. Through advances in supportive care, the mortality rate for patients with ALI/ARDS has decreased over time but still remains high [5,6]. Therefore, elucidating the pathogenesis of ALI/ARDS is necessary and may help with the development of novel therapeutic targets for ALI/ARDS.

The lung is exposed to a large number of potentially pathogenic microorganisms that are inhaled. To protect this vital organ against infection and inflammation, many defense mechanisms have been evolved in the lung to prevent the development of pulmonary infections. The secretion of endogenous cationic antimicrobial peptides by epithelial cells onto the airway surface represents an important component of immune defense in the lung [7-9]. Recent studies have demonstrated the importance of these antimicrobial peptides, such as defensins and cathelicidin, in the pulmonary immune system and in pulmonary diseases [10-12].

Hepcidin is a β-defensin-like antimicrobial peptide that is mainly produced by the liver. Hepcidin not only shows antimicrobial activity against Gram-positive bacteria, Gram-negative bacteria and yeasts, but also functions as a principal iron regulatory hormone [13-15]. Hepcidin binds to the iron export protein ferroportin and induces its internalization and degradation, which leads to decreased cellular iron export and increased intracellular iron retention [16,17]. Because iron is an essential nutrient for all organisms, hepcidin also restricts the iron available to invading microbes, thereby enhancing the host defense against pathogens [17-19]. Furthermore, hepcidin can modulate the lipopolysaccharide (LPS)-induced acute inflammatory response via the suppression of cytokine expression [20,21]. The multiple functions of hepcidin suggest its importance in the host immune response to infection and inflammation. Recent studies have reported that in addition to being produced mainly by hepatocytes, hepcidin is also expressed by other cells, such as airway epithelial cells (AECs) [22]. However, the role of AEC-derived hepcidin in pulmonary immune defense against infection and inflammation remains unknown.

In the present study, we generated a mouse model of AEC-derived hepcidin knockdown and investigated the impact of interfering with AEC-derived hepcidin on the pathophysiology of sepsis-induced ALI. We also explored the possible mechanisms involved.

## Materials and methods

### Adenovirus-mediated short hairpin RNA (shRNA)

As the mouse hepcidin gene *hepc1* performs similar biological actions as human hepcidin [23,24], we investigated *hepc1* in the present study. Small interfering RNA specific to the mouse *hepc1* (ACAGAUGAGACAGACUACAdTdT) was described previously [25], and we used this template to design the corresponding shRNA (ACCGACAGATGAGACAGACTACATTCATGAGATGTAGTCTGTCTCATCTGTCTTTTT) with minor modifications (Invitrogen, Shanghai, China). An adenovirus that expresses the *hepc1* shRNA or a nonspecific control oligonucleotide (GCCTAAGGTTAAGTCGCCCTCGCGAACGAAGGCGAGGGCGACTTAACCTTAGGTT) was prepared. Briefly, the double-strand shRNA oligo was cloned into pENTR/U6 vector using the BLOCK-iT U6 RNAi Entry Vector Kit (Life Technologies, Grand Island, NY, USA). The expression clone was then generated by recombination of pENTR/U6 entry construct and pAd/BLOCK-IT-DEST vector using BLOCK-iT Adenoviral RNAi Expression System (Life Technologies) according to the manufacturer’s instructions. After digested by endonuclease PacI (New England Biolabs, Ipswich, MA, USA), the recombinant adenoviral plasmid was transfected into 293A cells. The harvested adenovirus was concentrated and purified by CsCl gradient centrifugation. Viral titer was determined using Adeno-X Rapid Titer Kit (Clontech, Mountain View, CA, USA). The recombinant virus and control virus were named Ad-shHepc1 and Ad-shNeg, respectively.

### Animals and adenovirus treatment

C57BL/6 male mice aged six to eight weeks (Zhejiang Chinese Medicine University, Hangzhou, China) were used in this study. The mice were housed under standard care conditions. The animal studies were approved by the Animal Care and Use Committee of Zhejiang University (Reference No. 2014133; Hangzhou, Zhejiang Province, China) and handling of the animals were in accord with National Institutes of Health guidelines for ethical animal treatment.

To generate a mouse model of AEC-derived hepcidin knockdown, Ad-shHepc1 or Ad-shNeg (approximately 2.2 × 10^11^ viral particles) was intratracheally injected into the mice after anesthesia with chloral hydrate (400 mg/kg, intraperitoneally (i.p.)). To improve the knockdown efficiency, a second administration of the adenovirus was performed 72 hours later. Twelve days after the first injection, the mice were ready used in the experiments.

### ALI model

ALI was induced by polymicrobial sepsis with cecal ligation and puncture (CLP) surgery as described previously [26]. Briefly, after the abdominal wall was prepared with a 10% povidone-iodine solution, a 1-cm midline abdominal incision was made. The cecum was then exposed, isolated, and ligated with a 4-0 silk ligature just distal to the ileocecal valve to avoid intestinal obstruction. The puncture was performed twice with a 22-gauge needle. Then, the cecum was repositioned, and the incision was closed with a 4-0 sterile suture. Sham-operated mice were underwent the same procedure but without ligation and needle perforation of the cecum. At the end of the operation, the mice were resuscitated immediately by the subcutaneous administration of saline (1 ml/mouse) and allowed free access to food and water after awakening. In the experiment to evaluate the survival rate, the mice were monitored for survival every six to twelve hours until seven days post-CLP.

### Bronchoalveolar lavage fluid (BALF) analysis

Twenty-four hours after CLP, the mice were sacrificed via cervical dislocation. The chest cavity was opened via a midline incision. To perform the bronchoalveolar lavage, the lung was lavaged with 0.5 ml of chilled phosphate-buffered saline three times. In all cases, more than 90% of the total lavage volume was recovered. A 0.5-ml aliquot of BALF was used for the total cell counts and bacteriologic culture. The remaining BALF was centrifuged at 1000 g for five minutes, and the cell-free supernatant was stored at -80°C for further analysis.

#### Cell count

The total cell count in the BALF was determined using a hemocytometer.

#### Bacteriologic culture

The BALF was serially diluted in phosphate-buffered saline and inoculated on Luria Broth agar plates. After incubation at 37°C for 16 hours, visible colonies were counted and calculated as colony-forming units/ml of BALF.

#### Protein concentration assay

The total protein in the cell-free supernatant of the BALF was determined by using the BCA Protein Assay kit (Pierce, Rockford, IL, USA).

#### Cytokine measurement

The interleukin (IL)-6 level in the BALF was determined using an enzyme-linked immunosorbent assay kit according to the manufacturer’s instructions (Abcam, San Francisco, CA, USA).

### Lung wet/dry weight ratio

In separate groups the lung was excised, weighed, and then placed in an oven at 60°C for 72 hours to achieve the dry weight. The ratio of lung wet weight to dry weight was then calculated.

### Lung histology

The lung tissues from the mice were fixed in buffered 4% paraformaldehyde solution (pH = 7.4) for 24 hours, embedded in paraffin, and sectioned at a thickness of 4 μm. The histological examination was conducted in a blinded fashion after staining with hematoxylin and eosin.

### Quantitative polymerase chain reaction (qPCR)

For RNA extraction, alveolar macrophages were isolated by adhering bronchoalveolar lavage cells to plastic for one hour at 37°C in 5% CO_2_ [27]. Total RNA of the lung and liver tissues as well as the alveolar macrophages was extracted using the TRIzol™ Reagent. Reverse transcription was performed using 1 μg of total RNA with the Reverse Transcription System (Promega, Madison, WI, USA). The transcriptional level of hepcidin was quantified by real-time PCR using a standard SYBR™ Green PCR protocol on a CFX96 real-time PCR detection system (Bio-Rad Laboratories Inc., Hercules, CA, USA). The housekeeping gene β-actin served as an internal control, and the relative expression level of hepcidin was calculated using the 2^−ΔΔCT^ method.

### Immunohistochemistry

Immunohistochemical staining was performed following standard procedures. The formalin-fixed paraffin-embedded tissues were sliced into 4-μm-thick sections, deparaffinized, and rehydrated. For the hepcidin measurement in the alveolar macrophages, the cells were isolated by adhering bronchoalveolar lavage cells to coverslips at 37°C in 5% CO_2._ Then, the cells were fixed with 95% ethanol for 15 minutes. Antigen retrieval was performed in 10 mmol/L citric acid buffer (pH 6.0) for 10 minutes using a 750-W microwave. Endogenous peroxidase activity was blocked with 3% hydrogen peroxide in methanol for 15 minutes. After incubation with rabbit anti-mouse hepcidin antibody (1:400 dilution; Abcam) overnight at 4°C, the sections were washed in phosphate-buffered saline and incubated with a polymer horseradish peroxidase-conjugated secondary antibody (ZSGB-Bio, Beijing, China) for one hour. The sections were further incubated with Dako Liquid DAB Large-Volume Substrate-Chromogen System (DAKO, Glostrup, Denmark) and counterstained with hematoxylin. Negative controls were included in all assays by replacing the rabbit anti-mouse hepcidin antibody with nonimmune rabbit antiserum. The immunostaining was evaluated using an Olympus BX-50 light microscope (Olympus, Tokyo, Japan). The stain density was analyzed using the Image Pro-Plus 6.0 analysis system (Media Cybernetics Inc., Silver Spring, MD, USA), and the level of hepcidin was measured as the integral optical density.

### Western blot analysis

The protein concentrations in the lung homogenate or lysate of alveolar macrophages were detected using a BCA Protein Assay Kit (Pierce). The proteins (20 μg) were denatured by heating at 70°C for 10 minutes in 4 × nuPAGE LDs sample buffer (Life Technologies) and separated by nuPAGE Bis-Tris gel electrophoresis (Life Technologies). Then, the proteins were blotted onto polyvinylidene fluoride membrane (Millipore, Billerica, MA, USA). The membranes were blocked with 5% nonfat milk in Tris-buffered saline with 0.05% Tween-20 and incubated overnight with goat anti-ferroportin antibody (Santa Cruz Biotechnology, Dallas, TX, USA). The membranes were then washed with Tris-buffered saline with 0.05% Tween-20 three times for five to ten minutes each. After incubation with the related horseradish peroxidase-conjugated secondary antibody (Jackson ImmunoResearch Laboratories, Inc., West Grove, PA, USA), the membranes were visualized with the SuperSignal West Pico Chemiluminescent Substrate (Pierce). The signals were quantified using the Image J software by Wayne Rasband (National Institute of Health, Bethesda, Maryland, MD, USA). β-Actin served as a protein control.

### Iron determination

The iron content in alveolar macrophages and spleen was determined by Prussian blue staining using a commercially available kit according to the manufacturer’s instructions (Shanghai Yuanye Bio-Technology Co., Shanghai, China). The stain density in the spleen was analyzed using the Image Pro-Plus 6.0 analysis system as described above. For the iron measurement in the alveolar macrophages, the cells were isolated by adhering bronchoalveolar lavage cells to coverslips at 37°C in 5% CO_2._ Then, the cells were fixed with 95% ethanol for 15 minutes. The cells were considered positive with the presence of blue-colored granules within intact alveolar macrophages under a microscope. At least 200 random macrophages were counted, and the results are presented in terms of the percentage of positive cells.

The serum iron concentration was measured using atomic absorption spectroscopy [28].

### Phagocytosis assay

To investigate the phagocytosis function of alveolar macrophage, alveolar macrophages were isolated as described above. The cells were then incubated with fluorescent *Escherichia coli* (Life Technologies) for two hours. Fluorescence from the extracellular bacteria was quenched with 0.4% trypan blue. After three washes with phosphate-buffered saline, the fluorescence was observed under a fluorescence microscope (Olympus). The phagocytic index was quantified as number of fluorescent *E.coli* internalized by one macrophage cell counted in 10 random fields.

### Statistical analysis

The data are expressed as the mean ± standard deviation (SD) or median with range where applicable. The differences between the two groups were analyzed by the *t* test or Mann-Whitney *U* test. The survival curves were analyzed by the Kaplan-Meier log-rank test. The statistical analyses were performed using GraphPad Prism software 5.0 (GraphPad Software Inc., La Jolla, CA, USA) and SPSS 16.0 for Windows (SPSS Inc., Chicago, IL, USA). A two-tailed *P* value of less than 0.05 was considered to be statistically significant.

## Results

**Figure 1:**
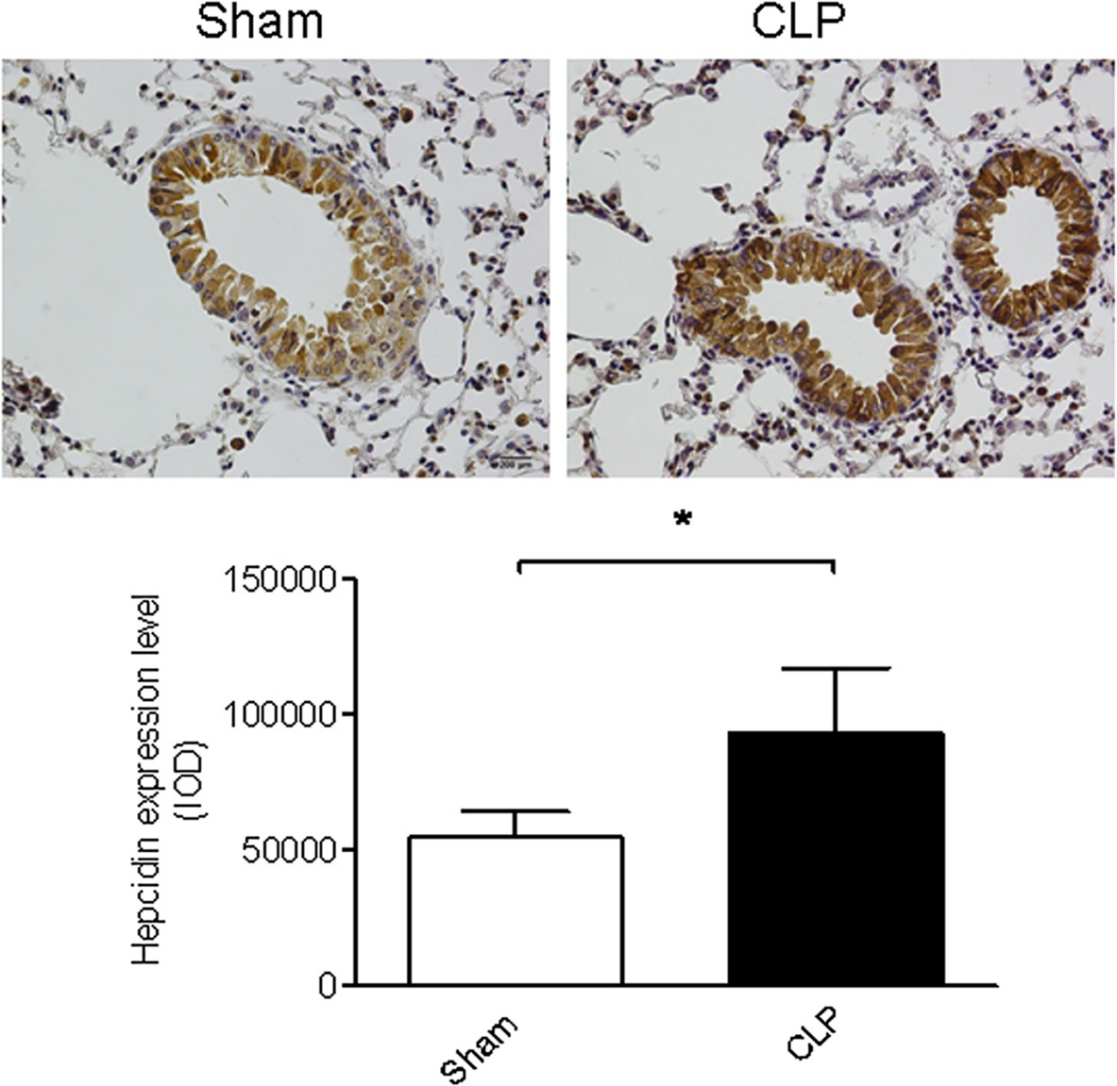
**The expression of hepcidin in airway epithelial cells (AEC) was upregulated after cecal ligation and puncture (CLP) surgery.** Hepcidin expression was examined by immunohistochemistry. A representative image is presented (magnification: ×400) and quantified data are shown. n = 6 mice/group. *P <0.005.

Hepcidin expression is modulated in response to infectious and inflammatory stimuli [14,17]. We first investigated whether the hepcidin expression level in the lung tissue changed during polymicrobial sepsis. Twenty-four hours after CLP surgery, the hepcidin level was significantly increased in the lung tissue, especially in the AECs (Figure 1). This finding indicates that hepcidin derived from AECs may play an important role in sepsis-induced ALI.

**Figure 2:**
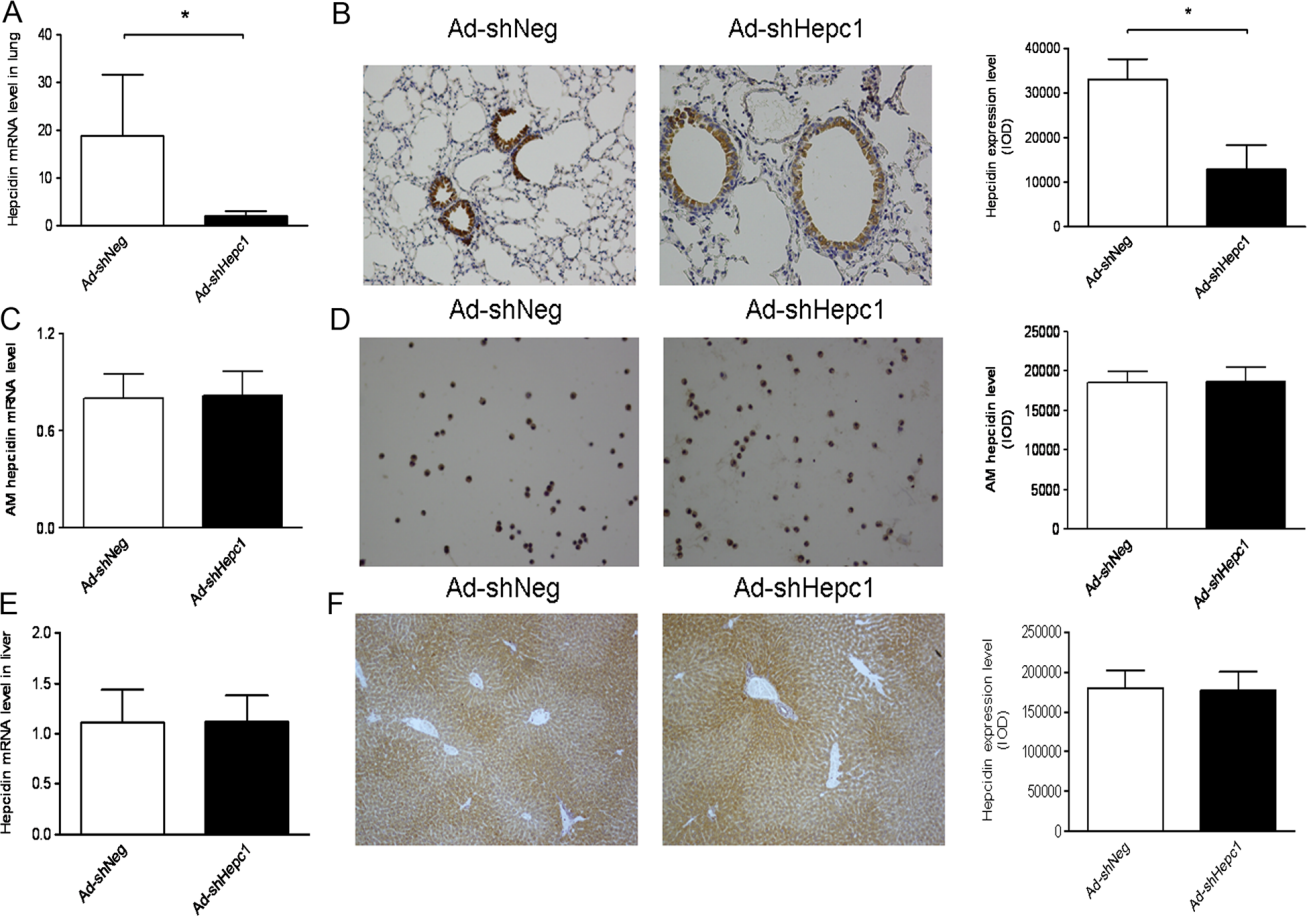
**Evaluation of hepcidin silencing** ***in vivo*** **after cecal ligation and puncture (CLP) surgery. (A, B)** Hepcidin expression in lungs was detected by quantitative polymerase chain reaction and immunohistochemistry. A representative image is presented (magnification: ×400), and quantified data are shown. n = 5 mice/group. *P <0.001. **(C, D)** Hepcidin mRNA and protein levels in alveolar macrophages were measured using quantitative polymerase chain reaction and immunohistochemistry. A representative image is presented (magnification: ×400), and quantified data are shown. n = 4 to 5 mice/group. **(E, F)** Hepcidin expression in livers was examined by quantitative polymerase chain reaction and immunohistochemistry. A representative image is presented (magnification: ×400), and quantified data are shown. n = 5 mice/group.

To confirm the role of hepcidin in ALI, Ad-shHepc1 or Ad-shNeg was administered to the mice via intratracheal instillation. Twelve days after administration of the adenovirus-mediated shRNA, the mice were subjected to an ALI model, and the hepcidin level in lung and liver were assessed at 24 hours after induction of ALI. As shown in Figure 2A and 2B, both the mRNA and protein levels of hepcidin in AECs was significantly reduced, whereas the hepcidin expression in alveolar macrophages (Figure 2C and 2D) and hepatocytes (Figure 2E and 2F) was not affected. These results demonstrated that in the current study the intratracheal administration of Ad-shHepc1 only silenced the hepcidin gene transcription in AECs, which was in accordance with previous studies that adenovirus-mediated intratracheal gene delivery specifically inhibited targeted gene expression in lung epithelial cells but not in alveolar macrophages and other organs [29,30].

**Figure 3:**
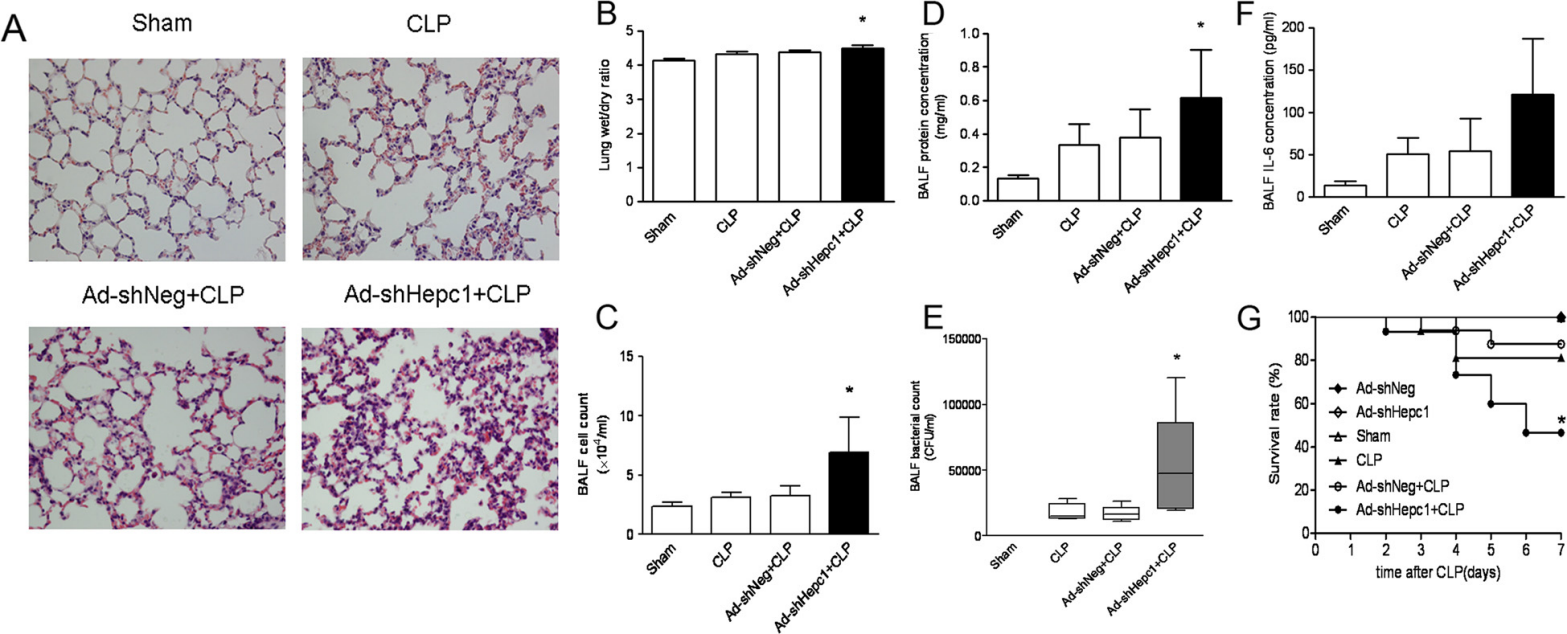
**The knockdown of hepcidin in airway epithelial cells worsened lung injury induced by cecal ligation and puncture (CLP) surgery. (A)** Hematoxylin and eosin stain of lung tissue sections. A representative image is presented (magnification: ×400). n = 5 to 6 mice/group. **(B)** Lung wet/dry weight ratio. n = 5 to 6 mice/group. *P <0.05 versus Ad-shNeg-treated group. **(C)** Total cell counts in BALF. n = 5 to 6 mice/group. *P <0.05 versus Ad-shNeg-treated group. **(D)** BALF protein concentrations. n = 5 to 6 mice/group. *P <0.01 versus Ad-shNeg-treated group. **(E)** Bacterial counts in BALF. n = 5 to 6 mice/group. *P <0.05 versus Ad-shNeg-treated group. **(F)** IL-6 concentrations in BALF. n = 5 to 6 mice/group. **(G)** Survival rate was monitored for seven days. n = 16 mice in the Ad-shNeg + CLP and CLP groups, and n = 15 mice in the other groups. *P <0.05 versus Ad-shNeg-treated group.

The lung injury was evaluated both in wild-type mice and adenovirus-treated mice at 24 hours after challenge with CLP or a sham operation. Histological characteristics of lung injury, including diffuse alveolar damage, infiltration of numerous leukocytes and interstitial edema, were observed both in the wild-type mice and Ad-shNeg-treated mice after CLP challenge. Knockdown of hepcidin in AECs led to more severe lung damage in the Ad-shHepc1-treated mice after CLP challenge (Figure 3A). Lung wet/dry weight ratios from the experimental mice further confirmed the histological findings (Figure 3B). The BALF analysis showed that the hepcidin knockdown mice had significantly higher cell counts and protein concentrations (Figure 3C and 3D). Of note, pulmonary bacterial colonization was much more severe in the Ad-shHepc1-treated mice (Figure 3E). However, the BALF IL-6 level in the hepcidin knockdown mice was not significantly different from that in the control mice (Figure 3F).

Moreover, the influence of disruption of the hepcidin gene in AECs on the outcome of ALI was further studied. CLP challenge caused a seven-day mortality of 18.75% in the wild-type mice, comparable to a mortality of 12.5% in the Ad-shNeg-treated mice. However, knockdown of hepcidin in AECs significantly increased the seven-day mortality after CLP surgery (53.33% versus Ad-shNeg-treated mice, *P* <0.05; Figure 3G).

**Figure 4:**
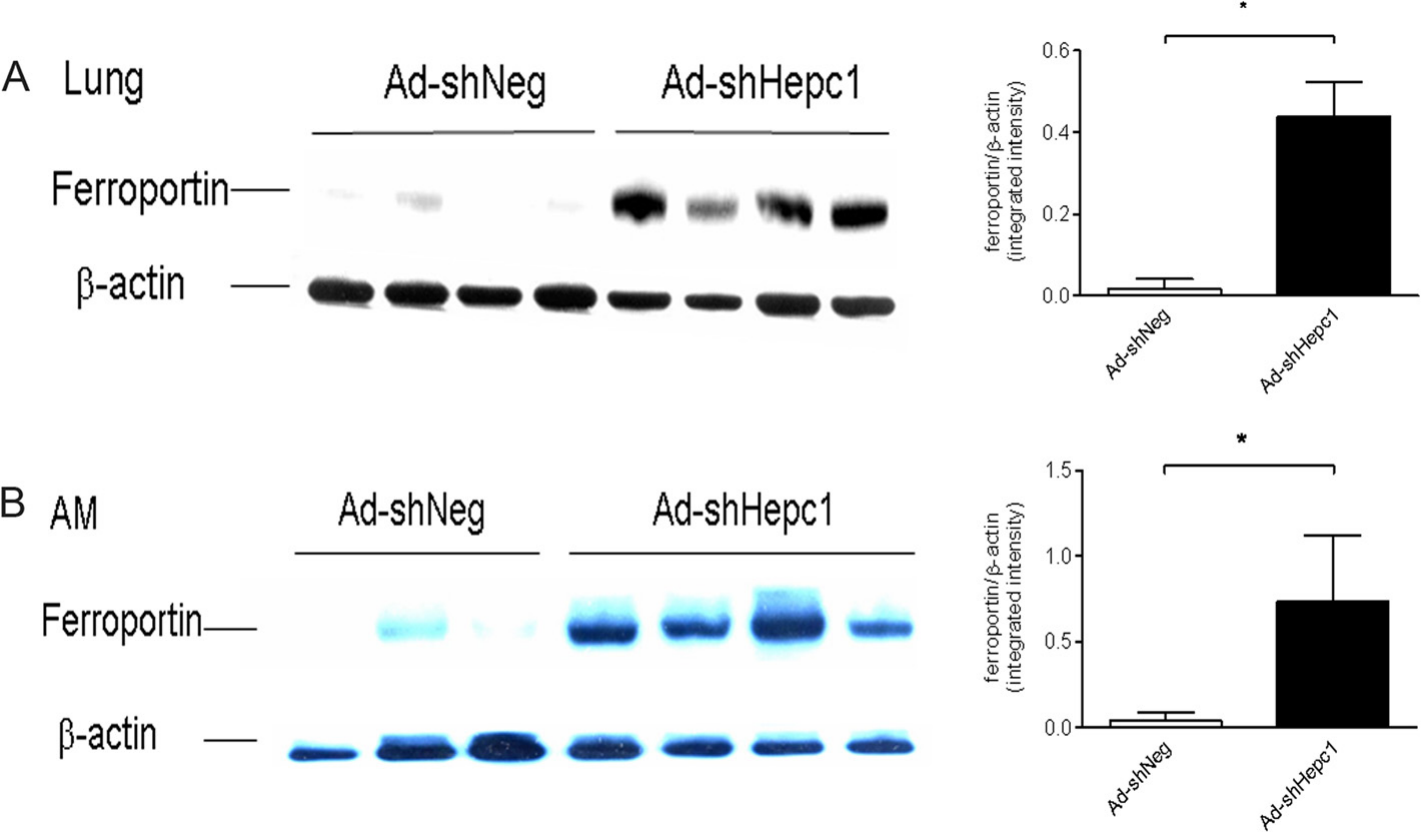
**The knockdown of hepcidin in airway epithelial cells affected pulmonary ferroportin after cecal ligation and puncture (CLP) surgery.** The ferroportin level was measured using Western blot analysis. Each lane represents one mouse. Quantified data are shown. **(A)** The ferroportin level in lung homogenates. *P <0.0001. **(B)** The ferroportin level in alveolar macrophages (AM). *P <0.05.

Hepcidin regulates iron metabolism by binding to ferroportin and causing its internalization and degradation. We therefore investigated the ferroportin levels in both whole lung tissue and alveolar macrophages. As expected, the ferroportin in the control mice was almost totally degraded, whereas a higher ferroportin level was observed in the hepcidin knockdown mice (Figure 4A and 4B).

**Figure 5:**
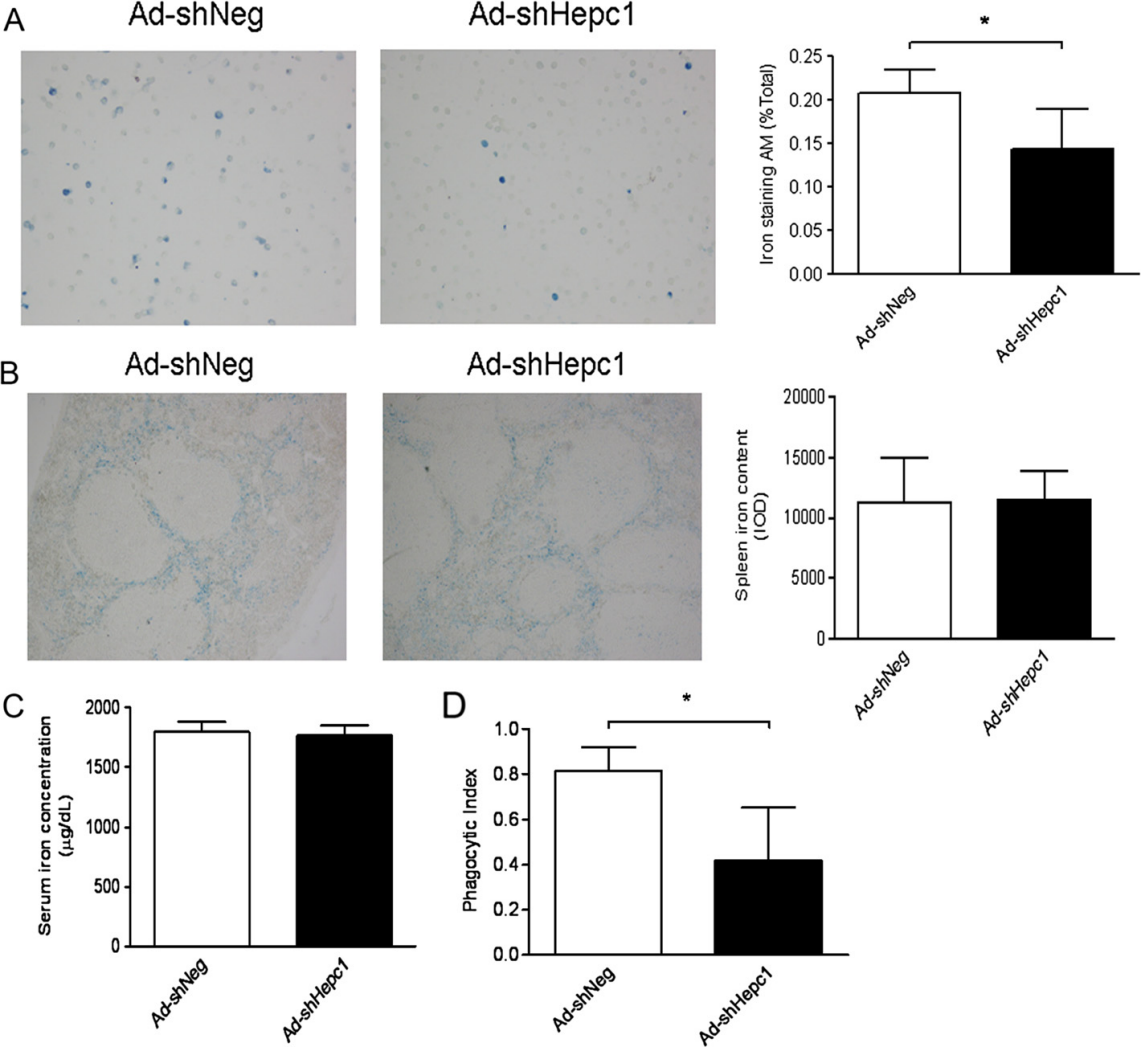
**The knockdown of hepcidin in airway epithelial cells altered intracellular iron metabolism and function of alveolar macrophages after cecal ligation and puncture (CLP) surgery. (A, B)** Iron levels in alveolar macrophages **(A)** and spleens **(B)** were evaluated by Prussian blue stain. A representative image is presented (magnification: ×400), and quantified data are shown. n = 5 to 6 mice/group. **(C)** Serum iron concentration was assayed using atomic absorption spectroscopy. n = 5 to 6 mice/group. *P <0.05. **(D)** Phagocytosis function of alveolar macrophages was accessed. n = 6 mice/group. *P <0.005.

We further asked whether hepcidin gene modification in AECs had an impact on local and systemic iron metabolism. Of note, the knockdown of hepcidin in AECs resulted in less iron retention in the alveolar macrophages (Figure 5A), whereas the iron contents in the spleen macrophages and serum iron concentration between the two groups showed no significant differences (Figure 5B and 5C).

Moreover, to link the intracellular iron content to the function of alveolar macrophages, we assessed the phagocytosis of alveolar macrophages, and found that the alveolar macrophages from Ad-shHepc1-treated mice showed less phagocytic ability than those from the control animals (Figure 5D).

## Discussion

In the current study, we found that the pulmonary hepcidin level was upregulated during polymicrobial sepsis. The knockdown of hepcidin in AECs aggravated the polymicrobial sepsis-induced lung injury and pulmonary bacterial infection and increased mortality. These pathophysiologic changes are at least partially related to the altered intracellular iron level and function of alveolar macrophages in the hepcidin knockdown mice.

Hepcidin is produced predominantly by hepatocytes. Its hepatic expression can be upregulated by iron overload and inflammation and suppressed by hypoxia and anemia [14,17]. A recent study reported that hepcidin is expressed in AECs in response to interferon-γ [22]. In the current study, an increased hepcidin level in AECs was observed during polymicrobial sepsis. Because the lung is the first vital organ that is adversely affected at the onset of sepsis [31], the elevated expression of hepcidin may protect the mice against lung injury. To support this hypothesis, knockdown of hepcidin in AECs exacerbated sepsis-induced lung injury (Figure 3). Considering that disruption of hepcidin has a deleterious effect, a low severity of CLP model was used in the current study. Since the underlying pathophysiology of sepsis is based on the severity grade of the CLP model, the increase in lung injury parameter such as wet/dry weight ratio was minuscule but significantly different.

Hepcidin is a master regulator of iron metabolism via its interaction with ferroportin. Hepcidin can trigger ferroportin polyubiquitination and induce ferroportin endocytosis [32,33]. In the lung, AECs express high levels of hepcidin and are the main source of local hepcidin production. Alveolar macrophages express ferroportin and are therefore target cells for hepcidin [22]. In the present study, knockdown of hepcidin in AECs impacted its interaction with ferroportin, and prevented degradation of the ferroportin protein in both alveolar macrophages and the lung, which consequently caused intracellular iron export into the pulmonary microenvironment. The elevated iron in the lung can be used not only for invading pathogen growth and replication, resulting in more virulent and persistent infections [18], but also to generate reactive oxygen species, leading to cell damage and lung injury [34,35]. On the other hand, a recent study showed that iron status could impact cytoskeleton rearrangement, which is important for the phagocytic process in macrophages [36]. Indeed, the alveolar macrophages from Ad-shHepc1-treated mice showed less phagocytosis ability than those from the control animals. Since liver is the major source of systemic hepcidin, in the current study liver hepcidin levels were not affected and circulating iron concentrations were comparable between the Ad-shNeg- and Ad-shHepc1-treated mice. Therefore, the function of circulating leukocytes should not be influenced. Although the inciting injury (CLP) is remote, when the bacteria circulating in the blood stream invaded the lung after CLP, the decreased phagocytosis function of the alveolar macrophages from the Ad-shHepc1-treated mice could result in bacterial accumulation in the lung. In addition, as hepcidin exhibits broad spectrum antimicrobial properties [37], the knockdown of hepcidin may contribute to more severe pulmonary infections and lung injuries.

Using primary human AECs and alveolar macrophages, Frazier *et al*. reported that the treatment with exogenous hepcidin did not affect ferroportin expression in the AECs and did not alter iron accumulation in both AECs and alveolar macrophages [22]. The contradictory findings between the current study and Frazier’s study may result from the difference between the *in vivo* model and *ex vivo* model used and the nature of hepcidin peptide. Moreover, limited by the sensitivity of the Prussian blue staining method, the iron content in the AECs was undetectable in this study. Because the role of AECs in pulmonary iron metabolism is much less than that of alveolar macrophages [22], the iron status in AECs may not play a major role in the pathophysiology of lung injury.

The production of cytokines and other inflammatory mediators at the site of injury is a feature of the pathogenesis of ALI, with IL-6 being one of the hallmarks [38]. However, the BALF IL-6 level in the AEC-specific hepcidin knockdown mice was not significantly different from that in the control mice. Previous studies found that low intracellular iron in macrophages could impair the translation of specific inflammatory cytokine transcripts, such as IL-6 [39,40]. Because alveolar macrophages are a major source of IL-6 production during lung injury, the decreased iron content in alveolar macrophages may compromise the local inflammatory response in the AEC-specific hepcidin knockdown mice.

## Conclusions

The current study explored the role of AEC-derived hepcidin in polymicrobial sepsis-induced ALI, which is at least partially related to the altered intracellular iron level and function of alveolar macrophages. These observations provide new insight into the pathogenesis of ALI/ARDS and might have therapeutic implications for ALI/ARDS.

## Key messages

- Knockdown of airway epithelial cell-derived hepcidin aggravated the polymicrobial sepsis-induced lung injury and pulmonary bacterial infection and increased mortality.
- Knockdown of hepcidin in airway epithelial cells led to reduced ferroportin degradation in lung and a low intracellular iron content in alveolar macrophages.
- These findings provide new insight into the pathogenesis of ALI/ARDS and might have therapeutic implications for ALI/ARDS.

## Abbreviations

AEC: airway epithelial cell
ALI: acute lung injury
ARDS: acute respiratory distress syndrome
BALF: bronchoalveolar lavage fluid
CLP: cecal ligation and puncture
IL: interleukin
LPS: lipopolysaccharide
qPCR: quantitative polymerase chain reaction
shRNA: short hairpin RNA
